# Magnetic resonance imaging in exertional compartment syndrome of the forearm: Case-based pictorial review and approach to management

**DOI:** 10.4102/sajr.v22i1.1284

**Published:** 2018-04-18

**Authors:** Bishum Rattan, Shalendra K. Misser

**Affiliations:** 1 Lake Smit and Partners Inc., Durban, South Africa; 2 School of Health Sciences, University of KwaZulu-Natal, South Africa

## Abstract

Exercise-related limb pain poses a management dilemma to the clinician. The term ‘chronic exertional compartment syndrome’ (CECS) (previously known as ‘anterior tibial syndrome’) refers to a condition characterised by exercise-induced pain in one or more muscle groups and is more commonly seen in the lower limbs. Much less has been reported about the upper limbs where the muscular compartments are variably noted to be involved. A high index of clinical suspicion should therefore be maintained to avoid missing the diagnosis. Although commonly noted in athletes, CECS can occur in any age group with any level of exercise activity. In addition, there is no age predilection and the syndrome may be bilateral. The exact prevalence is not known as many athletes modify their training methods, thus delaying or avoiding medical assistance and imaging. The pathophysiology of compartment syndrome is complex. In this review of the syndrome, we describe the cycle of intracellular events leading to CECS and the eventual destruction of muscle. There is considerable overlap with the many possible causes of limb pain. Even the most experienced clinicians experience some difficulty in making this diagnosis of CECS, but with increasing awareness of this entity and availability of good-quality magnetic resonance imaging to confirm the suspicion, upper limb CECS is being more commonly diagnosed and patients more timeously managed.

## Definition

Chronic exertional compartment syndrome is a debilitating and painful condition that results from abnormally increased pressure in a muscular compartment, enclosed by relatively non-compliant fascia, where increase in interstitial pressure compromises the vascular supply to the contents of the space. This leads to ischaemia, eventual muscle necrosis and nerve death. The predisposing cause is repeated activity involving specific muscle groups and is most commonly encountered with certain types of sport and less often associated with some vocations.

## Pathophysiology

**FIGURE 1:**
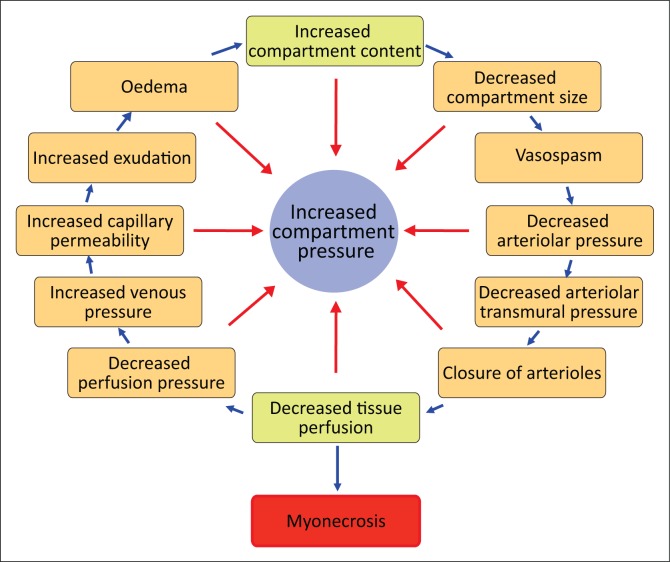
Algorithm detailing the pathophysiology of compartment syndrome, based on the theory of Matsen.

The pathophysiology of compartment syndrome is complex. The common prerequisite is a soft tissue structure (usually fascia) that prevents muscle expansion when muscle is exposed to increased fluid volume. Increased compartment pressure from either internal or external injury causes a decreased perfusion gradient between arteriolar and venous pressures and as a result, decrease in local tissue perfusion. Decreased tissue perfusion results in further tissue insult, greater capillary leakage and further increment in intra-compartmental pressure. This causes a vicious cycle (shown in Figure 1)^1^ of progressive cellular ischaemia, leading to further capillary leakage, swelling and increasing compartment pressure. In all cases, the final common pathway is cellular anoxia.

Ongoing ischaemia eventually (if not relieved by fasciotomy or prolonged rest as in the case of exertional compartment syndrome) leads to cell death and lysis of the myocyte. Degradative enzymes are activated and released into the interstitial tissues causing further tissue necrosis. The extent of muscle injury depends on the duration of ischaemia and the metabolic rate of the tissue.

## Types of compartment syndromes

Acute compartment syndrome^2^ is a medical emergency usually caused by injury and can lead to permanent muscle damage. In the acute setting, compartment syndrome may arise from extrinsic or intrinsic injuries.

Chronic exertional compartment syndrome (CECS) is not a medical emergency and is most often caused by long-standing athletic exertion. This is most commonly encountered in athletes with repetitive, severe stress involving muscle groups used for a specific action during sport. Fortunately, chronic compartment syndrome less likely results in the severe complications of acute compartment syndrome, namely, myonecrosis, atrophy, disability and deformity; however, chronic pain is a constant debilitating phenomenon which can be relieved, pending a definitive diagnosis.

The causes of acute and chronic compartment syndrome are listed in Table 1.^1^

**TABLE 1:** Causes of compartment syndromes.

Intrinsic injuries	Extrinsic injuries	Causes of CECS
Supracondylar or radial head fractures in a child Distal radial fractures in an adult Blunt soft tissue trauma Penetrating trauma Vascular injury or trauma reperfusion Burns Animal or insect bites Infection Bleeding disorders	Compressive casts Burn eschars Crush injury	Rowing Cycling Motocross Cricket Bodybuilding Military

CECS, chronic exertional compartment syndrome.

## Diagnosis or differential diagnosis

The cornerstone of diagnosis is based on a detailed history, high index of clinical suspicion and careful physical examination. Even in the most astute clinician’s hands, the distinction between the different medical causes may be difficult, given the overlap of clinical features. There are several distinct entities that can present with limb pain (listed in Box 1) and these may be clinically indistinguishable from CECS.^3^

Box 1 The differential diagnosis for limb pain.

**Differential diagnosis**

- Chronic exertional compartment syndrome
- Tibial stress injury or periostitis (lower limb)
- Common flexor or extensor tendinosis (upper limb)
- Venous thrombus
- Arterial disorders:
  - entrapments
  - dissections
  - injuries
  - chronic post-traumatic haematomas
  - claudication or insufficiency
- Myopathies
- Bone or soft tissue tumours
- Stress fractures
- Delayed onset muscle soreness
- Peripheral neuropathy

Intra-compartment pressure can be measured clinically via a slit catheter or similar pressure-transducing device. This method has been the gold standard of diagnosis of CECS; however, such pressure measurements are not without challenges.^4^ Ideally, measurements should be obtained before, during and after exercise. The measurement is multifocal, painful, invasive and sometimes not available in some centres. In addition, there are potential risks of infection and bleeding. The compartment pressure measurement is not standardised and there is inter-observer variability in testing which impacts decision-making with regard to proceeding to surgery.^4^

Consequently, diagnostic imaging, especially MRI evaluation, is a useful tool in diagnosing CECS. Over the last two decades, MRI has been shown to have a very high accuracy in confirming the diagnosis of CECS.^5^ Ultrasound has also been shown to be of value in the clinical work-up of patients with suspected CECS,^6^ especially of the lower limbs. The inter-observer variability as well as the reduced sensitivity and specificity of ultrasound lowers diagnostic efficacy when compared to MRI. In addition, it is more difficult to demonstrate, in a reproducible manner, the dynamic muscular changes on ultrasound.^7^ These pathophysiological changes are more clearly shown on MRI imaging sequences recorded before and after exercise.

## Clinical presentation of chronic exertional compartment syndrome

Patients with CECS typically complain of pain that begins within the first 30 min of starting an activity.^8^ Burning, cramping or aching pain progresses as the activity is continued. Typically, the pain increases to a level of discomfort where the patient can no longer continue or where it adversely affects the patient’s performance.^1^ The key clinical features of CECS are listed in Box 2. ^8,9,10,11^ Activities associated with exertional compartment syndrome of the upper limb include sports such as rowing, cycling, motorcycle riding, cricket, body building (particularly those supplementing with creatine and anabolic steroid) or repeated episodes of manual labour requiring a prolonged grip or pinch.^9^ In the United States, CECS was documented in military recruits, especially those involved in battlefield training.^10^ In most instances, the pain and induration resolve spontaneously on cessation of the activities.^1^

Box 2 Clinical features of chronic exertional compartment syndrome.

**Features**

- Swelling or tightness of compartment
- Progressive pain
- Inappropriate and uncontrolled pain
- Severe pain at rest or passive stretching
- Pallor or cyanosis
- Hyperesthesia or paraesthesia
- Exertional pain with gradual recovery on rest
- Paralysis (full recovery is rare)

## Forearm compartments and anatomy

The forearm can be divided into the volar and deep compartments, as listed in Box 3^1^. The volar compartment can be divided into the superficial and deep groups and the dorsal compartment can be divided into the mobile wad, the extensor group and the anconeus. It is delineated proximally by the lacertus fibrosus and pronator teres and distally by the carpal tunnel.

Box 3 The anatomical divisions of the forearm compartments.^1^

**Table:** 

Volar compartment	Dorsal compartment
**Superficial** Flexor carpi radialis (FCR) Palmaris longus (PL) Pronator teres Flexor carpi ulnaris (FCU) Flexor digitorum superficialis (FDS)	**Mobile wad** Brachioradialis (Br) Extensor carpi radialis longus (ECRL) Extensor carpi radialis brevis (ECRB)
**Deep** Flexor digitorum profundus (FDP) Flexor pollicis longus (FPL) Pronator quadratus Anterior interosseous nerve and artery	**Extensor** Extensor digitorum communis (ED) Extensor carpi ulnaris (ECU) Extensor pollicis longus (EPL) Abductor pollicis longus (APL) Extensor pollicis brevis (EPB) Supinator muscles (S) Posterior interosseous nerve

Except for the strong interosseous membrane, forearm fascial planes often incompletely separate compartments, allowing some communication between muscle groups. In the setting of forearm compartment syndrome, surgical fasciotomy of one muscle compartment may thus be sufficient to decompress another.^1^

**FIGURE 2:**
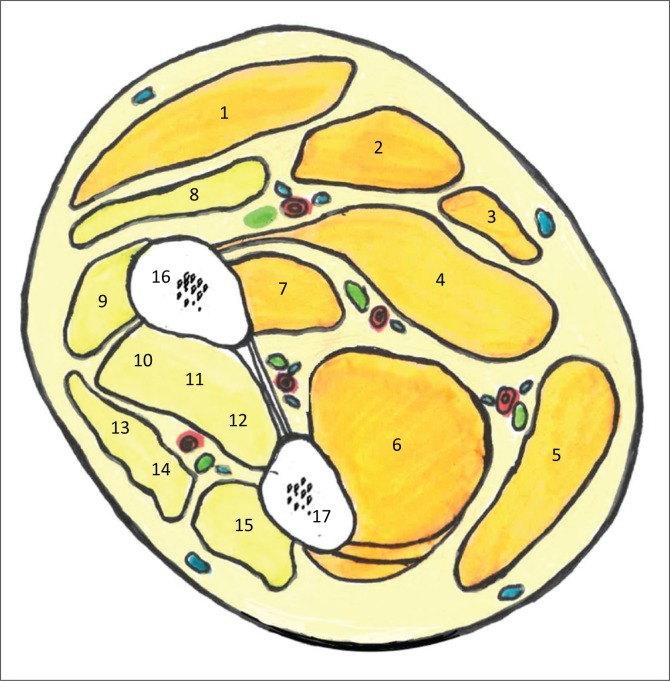
Cross-sectional diagram of the mid forearm delineating the compartments of the volar and dorsal forearm: (1) Brachioradialis; (2) Flexor carpi radialis (FCR); (3) Palmaris longus; (4) Flexor digitorum superficialis; (5) Flexor carpi ulnaris; (6) Flexor digitorum profundus (FDP); (7) Flexor pollicis longus (FPL); (8) Extensor carpi radialis longus; (9) Extensor carpi radialis brevis; (10) APL; (11) Extensor pollicis brevis; (12) Extensor pollicis longus (EPL); (13) Extensor digitorum communis (ED); (14) Extensor digiti minimi; (15) Extensor carpi ulnaris; (16) Radius; (17) Ulna.

The anatomical considerations of chronic compartment syndrome in the lower limb have been well described in the literature. In the forearm, the volar and dorsal compartments are interconnected, unlike the leg compartments. Several anatomical structures however limit the dorsal and volar compartments. The interosseous membrane, the radius and ulna separate the volar and dorsal compartments. The antebrachial fascia limits anteriorly the volar compartment and posteriorly the dorsal compartment, as shown in Figure 2.^1^

The thick aponeurosis of the FDP separates the superficial from the deep volar compartment. The median and ulnar nerves are located lateral and medial, respectively, relative to the FDP aponeurosis. Interconnections between the superficial and deep volar compartments are not sufficient to prevent a large increase in pressure during continuous muscular contraction, especially within the flexor compartments. Thus, chronic forearm compartment syndrome has been described in strength training, long distance rowing and motorcycle activities in which flexor muscle contraction is continuous, to control the brake lever and keep the motorbike on the road.^11^

## Magnetic resonance imaging

The MRI examination is often used as a problem-solving technique in certain circumstances and in patients refusing intra-compartmental measurements or in patients where such invasive measurements are contraindicated, for example, coagulopathies. It has been shown that MRI findings are comparable to that of intra-compartmental pressure measurement, and this investigation is increasingly being used in the diagnostic evaluation of CECS.^12^

## Protocol

Patients are imaged before and after pain-inducing exercise with contrast, if not contraindicated.^5^ The basic sequences utilised are listed in Box 4; however, the protocol may vary at different institutions. The axial and coronal scan planes are used. In most instances, the axial plane is preferred allowing for easier identification, and more accurate and reproducible labelling of the various muscle groups.

Box 4 The magnetic resonance imaging pre- and post-exercise protocol.

**Exercise protocol:**

- T1-weighted sequence for anatomy
- T2-weighted sequence with fat suppression before and after exercise
- Short tau inversion recovery sequence (STIR)
- Pre- or post-contrast T1-weighted sequence
- FIESTA imaging – to exclude vascular aetiology, sometimes used

FIESTA, Fast Imaging Employing Steady-state Acquisition.

## Findings

**FIGURE 3:**
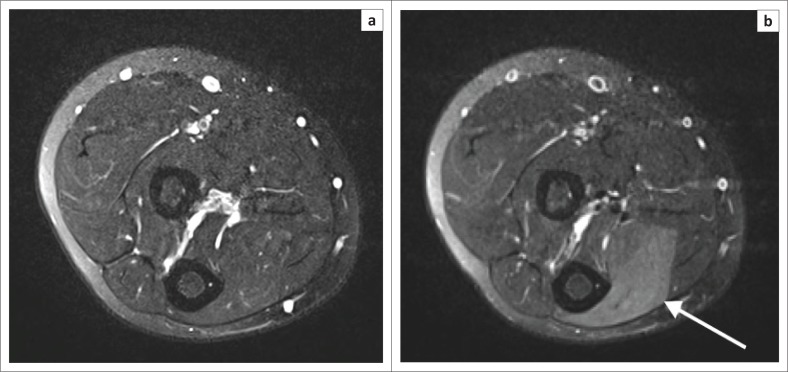
(a) Axial fat-suppressed T2-weighted magnetic resonance imaging of the right forearm, pre-exercise demonstrates iso-intensity of muscle groups. Note the normal calibre of intramuscular vessels; (b) Axial fat-suppressed T2-weighted magnetic resonance imaging of the right forearm, post-exercise – white arrow showing hyperintensity of the flexor digitorum profundus muscle and effaced intramuscular vessels.

A change in signal and sometimes an increase in volume on T2W imaging in an isolated compartment associated with increased activity, support the diagnosis of exercise-induced compartment syndrome. Magnetic resonance imaging is a valuable adjunct to the clinical diagnosis as it serves to identify the affected compartment and provide a road map prior to surgery, if indicated. This is particularly important where there is involvement of more than one muscle compartment. Interstitial haemorrhage can be demonstrated on gradient echo sequences and depending on the age of the haemorrhage may show hyperintensity on T1-weighted sequences and hyper- or hypo-intensity on T2-weighted sequences. Box 5 outlines the MRI findings in CECS. These features are demonstrated in two case studies below (Figures 2 and 3).

Box 5 The magnetic resonance imaging features of upper limb compartment syndrome.

**Features:**

- Generally confined to a single muscular compartment
- Sometimes involvement of more than one compartment
- Typical anatomic distribution
- May reveal interstitial haemorrhage
- Loss of the normal muscular septation
- Muscular hyperintensity (oedema) on fluid-sensitive sequences
- There may or may not be swelling or increase in volume
- Sometimes hyperintensity may be inhomogeneous and not confluent
- Post-contrast imaging may reveal compartmental enhancement
- Myonecrosis is a late phenomenon
- Chronic phase – fibrosis, calcifications, cystic changes
- Late stage – chronic atrophy

Axial post-gadolinium, fat-saturated, T2-weighted turbo spin echo sequence, performed in a professional rower, as shown in Figure 3, undertaken before (Figure 3a) and after (Figure 3b) exercising the right forearm. Note the hyperintensity demonstrated within the proximal muscle belly of the right FDP muscle after exercising. There is marked swelling of the FDP muscle and associated effacement of the intramuscular vessels on the post-exercise image, indicative of elevated compartment pressure. It has been reported that in untrained individuals, intramuscular hyperintensity may be seen on the post-exercise MRI studies, especially when irregular or eccentric training is practiced.^13,14^ This can mimic CECS and should be correlated with direct compartment pressure measurement.^12^

**FIGURE 4:**
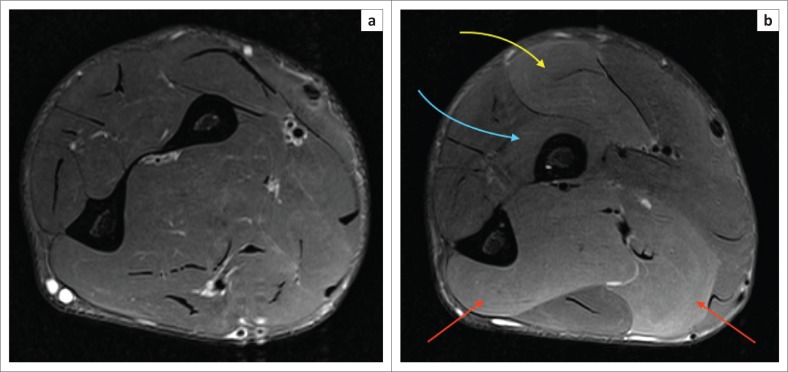
(a) Axial fat-suppressed, T2-weighted magnetic resonance imaging of the left forearm, pre-exercise demonstrates iso-intensity of muscle groups. Note again the normal calibre of intramuscular vessels; (b) Axial fat-suppressed, T2-weighted magnetic resonance imaging of the left forearm, post-exercise. Arrows show hyperintensity of the muscle groups with effaced intramuscular vessels. The yellow arrow points to the flexor carpi radialis (FCR); the blue arrow points to the flexor pollicis longus (FPL); the red arrow points to the extensor compartment, including abductor pollicis longus (APL), extensor pollicis longus (EPL) and extensor digitorum communis (ED).

Chronic exertional compartment syndrome of the forearm can involve multiple muscle compartments and variably involve individual muscles in a compartment, whilst sparing others. This is well demonstrated in the MRI images of an elite endurance athlete in Figure 4. The pre-exercise, fat-suppressed, T2-weighted image of the left forearm (Figure 4a) shows moderate swelling of the muscles of both the flexor and extensor compartments with iso-intensity. This patient had pain in his forearm, even at rest, which progressed to excruciating pain on exercise. The hyperintensity involving multiple muscle groups of the flexor and extensor compartments is demonstrated in Figure 4b.

## Management

In most cases prior to the firm diagnosis of CECS, some degree of conservative management is suggested. This includes non-steroidal anti-inflammatory drug (NSAID) therapy and activity modification through decreasing the duration and intensity of workouts. These may not offer significant relief. Surgical management is definitive and elective fasciectomy or fasciotomy is the offered treatment option.^15^ It is noted that surgical treatment is not without risks of complications, which include infection, scarring, permanent nerve damage and numbness.^12,15^

**FIGURE 5:**
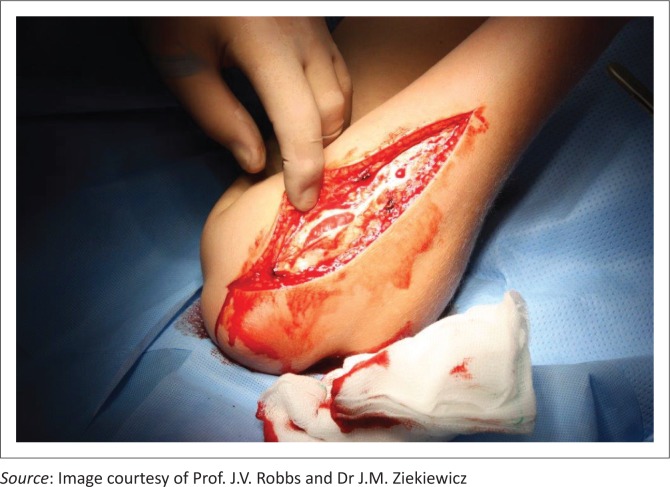
Open fasciotomy of the left forearm exposing the intermuscular septum and fascia.

There have been many surgical incisions of the forearm described with most being long and extensile, hence, most incisions can be used to decompress tense forearm compartments. Figure 5 demonstrates open fasciotomy, exposing the intermuscular septum and fascia. Generally, these incisions are left open, hence it is preferred to use one that minimises the exposure of the neurovascular structures and can be extended (if required) to the medial elbow proximally or to the carpal tunnel distally.

**FIGURE 6:**
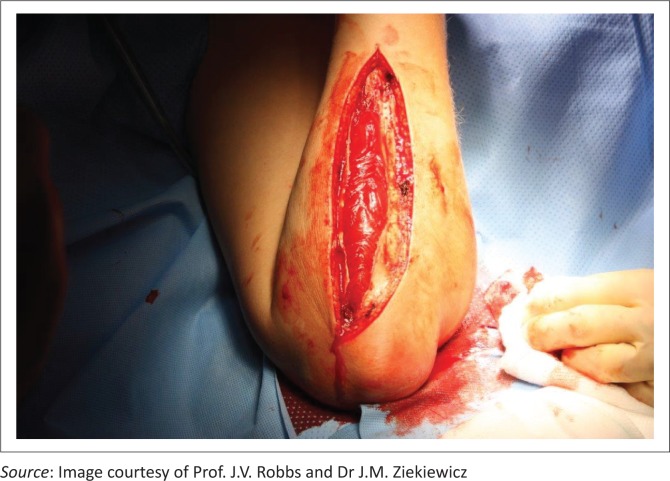
Completion fasciotomy.

On the volar aspect, the antebrachial fascia is released longitudinally from the lacertus fibrosus to the wrist flexion crease to release the superficial flexor compartment and an incision is made on the ulna side to release the deep flexor portion. During dissection, some of the muscular compartments may appear pale suggesting compromise, hence additional release of the epimysium is recommended. If the epimysium is not released, this muscle belly may run the risk of reperfusion injury which will lead to further muscle damage. Figure 6 shows a completion fasciotomy and partial fasciectomy releasing the tense compartment.

Similarly, the extensor compartment is released through a midline dorsal longitudinal incision extending from the lateral epicondyle to the distal radioulnar joint. This allows for release of the mobile wad and the extensor compartment. Once the swelling has subsided, either primary closure or skin grafting can be used to close the wound. In addition, physiotherapy or movement should be commenced immediately following surgery to promote active and passive ranges of movement.

## Conclusion

Chronic exertional compartment syndrome has characteristic MRI features and the radiologist plays a key role in facilitating a correlation between clinical presentation and confirmation of the diagnosis. With increasing awareness of this clinical entity and availability of good-quality MRI studies to confirm the suspicion, upper limb CECS is being more commonly diagnosed and patients more timeously managed.
